# Comparative Biology of Cycad Pollen, Seed and Tissue - A Plant Conservation Perspective

**DOI:** 10.1007/s12229-018-9203-z

**Published:** 2018-07-05

**Authors:** J. Nadarajan, E. E. Benson, P. Xaba, K. Harding, A. Lindstrom, J. Donaldson, C. E. Seal, D. Kamoga, E. M. G. Agoo, N. Li, E. King, H. W. Pritchard

**Affiliations:** 1 Royal Botanic Gardens, Kew, Wakehurst Place, Ardingly, West Sussex RH17 6TN UK; 2 Present Address: The New Zealand Institute for Plant & Food Research Ltd, Private Bag 11600, Palmerston North, 4442 New Zealand; 3 Damar Research Scientists, Damar, Cuparmuir, Fife, KY15 5RJ UK; 4 South African National Biodiversity Institute, Kirstenbosch National Botanical Garden, Cape Town, Republic of South Africa; 5 Nong Nooch Tropical Botanical Garden, Chonburi, 20250 Thailand; 6 Joint Ethnobotanical Research Advocacy, P.O.Box 27901, Kampala, Uganda; 7 De La Salle University, Manila, Philippines; 8 Fairy Lake Botanic Garden, Shenzhen, Guangdong People’s Republic of China; 9 UNEP-World Conservation Monitoring Centre, Cambridge, UK

**Keywords:** Threatened plants, Germination, Storage, In vitro technology, Biotechnology

## Abstract

Cycads are the most endangered of plant groups based on IUCN Red List assessments; all are in Appendix I or II of CITES, about 40% are within biodiversity ‘hotspots,’ and the call for action to improve their protection is long-standing. We contend that progress in this direction will not be made until there is better understanding of cycad pollen, seed and tissue biology, which at the moment is limited to relatively few (<10%) species. We review what is known about germplasm (seed and pollen) storage and germination, together with recent developments in the application of contemporary technologies to tissues, such as isotype labelling, biomolecular markers and tissue culture. Whilst progress is being made, we conclude that an acceleration of comparative studies is needed to facilitate the integration of in situ and ex situ conservation programmes to better safeguard endangered cycads.

## Introduction

Cycads are gymnosperms and current cycad families are considered to be remnants of the most ancient group of seed-bearing plants, with origins of the cycad crown dating to c. 200 million years ago (MYA) (Nagalingum et al., 2011) and c. 230 MYA (Salas-Leiva et al., 2013). During the Jurassic period (c. 200–145 MYA), cycads are thought to have been relatively common in the world’s flora. The evolutionary age estimates and diversification dynamics of cycads depend on the dating method used. Cycad genera either diversified in the Palaeogene (66–23 MYA), and had two diversification rate shifts, or during the Neogene (23–2.6 MYA), with four rate shifts accounting for each of the four richest genera: *Cyacas, Zamia, Encephalartos* and *Macrozamia* (Condamine et al., 2015). However, new evidence suggests that today’s living species are not much older than ~12 million years, and are thus not living fossils per se (Litz et al., 2005; Nagalingum et al., 2011; Salas-Leiva et al., 2013). Nonetheless, current cycad species are viewed as evolutionary relicts in the sense that they are surviving representatives of once diverse or abundant groups, having persisted with little morphological change (Nagalingum et al., 2011). They are generally regarded as extremely slow-growing species, with a mean growth rate varying from a maximum of ca. 10–15 cm per year, in a few taller-growing species, to ca. 1 cm per year, in many forms with more extreme, xerophytic adaptations and dwarf species (Norstog & Nichols, 1997).

Cycads (literally Cycadales) comprise three families (Cycadaceae, Stangeriaceae and Zamiaceae) and consist of a total of 10 accepted genera and 348 accepted species, divided as follows: *Bowenia* (2), *Ceratozamia* (30), *Cycas* (114), *Dioon* (15), *Encephalartos* (65), *Lepidozamia* (2), *Macrozamia* (41), *Microcycas* (1), *Stangeria* (1), and *Zamia* (77) (Calonje et al., 2017; Osborne et al., 2012). The species are distributed in warmer areas of North and South America, Africa, Asia and Australia. Interestingly, cycad species have a near symmetrical latitudinal distribution north and south of the equator, with peaks at latitudes c. 27° S and 18° N (Fragniere et al., 2015). Species richness is relatively low at the equator, with no species in temperate latitudes >40°. The Caribbean and northeast Australia represent the two most diverse floristic regions, with c. 70 species each; while 38 *Encephalartos* species and 31 *Cycas* species are found in the Usambara–Zululand and Indochinese regions respectively (Fragniere et al., 2015). Cycads occur at relatively low elevations (mean of 565 m); only four species reach above 2000 m (Fragniere et al., 2015).

In many instances, individual species tend to exist as small, isolated populations in transformed habitats. Such biogeographical restrictions have contributed to a high estimate for the risk of cycad extinction based on the IUCN Red List categorisation which relates to the area of occupancy (AOO) and extent of occurrence (EOO). Donaldson (2003) reported 62% of cycads as being threatened with extinction, making them the most threatened group of plant species on Earth. This estimate has been confirmed recently with an analysis of the 2016 IUCN Red List (Table 1), showing that c. 60% of cycad species assessed were either Critically Endangered (CR), Endangered (EN) or Vulnerable (VU) (Fragniere et al., 2015; Marler & Marler, 2015; IUCN, 2016). Primarily, CR species are from the genera *Encephalartos*, *Zamia* and *Cycas* (Table 1). In comparison, it has been estimated that just over one fifth of all plant species might be threatened with extinction, and the habitat with the most threatened species is overwhelmingly tropical rain forest (Brummitt et al., 2015). Risks of extinction in the natural habitat are exacerbated by complex reproductive cycles and dependencies on other species for N-fixation, pollination and seed dispersal.

**Table 1:** Cycad species in the three families assigned Critically Endangered (CR), Endangered (EN) or Vulnerable (VU) status

Family	Genus	Conservation status of species: CR / EN / VU (n)	Total threatened species (n)
Cycadaceae	*Cycas*	11 / 16 / 30	57
Stangeriaceae#	*Stangeria*	0 / 0 / 1	1
ZamiaceaeΦ	Encephalartos*	17 / 10 /15	42
	*Ceratozamia*	7 / 12 / 3	22
	*Dioon*	0 / 5 /6	11
	*Macrozamia*	0 / 8 / 9	17
	*Microcycas*	1 / 0 / 0	1
	*Zamia*	16 / 12 / 12	40
		Σ	191 (+4)*

A total of 303 species were assessed. Data collated from Fragniere et al. (2015) and IUCN (2016)

# no species in the genus *Bowenia* are listed as CR, EN or VU

Φ no species in the genus *Lepidozamia* are listed as CR, EN or VU

*plus four species that are extinct in the wild (*Encephalartos brevifoliolatus*, *E. nubimontanus*, *E. relictus* and *E. woodii*)

Cycads are popular and charismatic species for collectors and landscaping, and this has resulted in an increase in wild-sampling, which has also contributed to rarity. Excessive and illegal harvesting of mature cycads from natural populations has caused colony destruction and the extinction of several wild populations. Threats to wild cycads also include habitat destruction for farming, mining and urban development, habitat modification, traditional use (medicinal), invading alien vegetation and, increasingly, climate change and phyto-pathological threats.

In addition to biogeographical limitations, cycads are vulnerable to anthropogenic activities and require particular conservation attention. An example of the urgency assigned to the conservation of cycads is that 6% of the 120 species listed by the State Forestry Administration (SFA) of China for urgent integrated (in situ and ex situ) conservation intervention are *Cycas* species, e.g., *C. chanjiangensis, C. debaoensis, C. dolichophylla, C. fairylakea, C. hongheensis, C. szechuanensis and C. taiwaniana* (Wade et al., 2016).

The question then arises: What type of strategies are required to improve the conservation status of cycads? The concept of taking an integrated conservation approach for cycad species was presented at the 9th International Conference of Cycad Biology (Pritchard et al., 2011). As for other ‘plant species with extremely small populations’ (PSESP; Ma et al., 2013; Wade et al., 2016), a range of innovative interventions are required to ensure the integrated conservation of highly threatened species (Pritchard et al., 2014; Wade et al., 2016). These include: pollen collection, storage and use to artificially pollinate ‘cones’ in the field so as to enhance quality seed output and potentially widen the genetic diversity of cycad populations; seed treatments for germination; and tissue use for provenance assessment and propagation. However, such actions depend on a far better understanding of many aspects of the biology of cycad pollen, seed and tissues.

## Pollen and Reproductive Biology

Each cycad genus has unique reproductive and vegetative characteristics. However, all cycads are dioecious where each individual plant is strictly male or female throughout their life cycle. The pollen (on the male plant), and also the ovules (of the females) are produced on sporophylls which are aggregated as cones. Cycad cones vary in size, with female cones ranging from <5 cm long to 1 m long, and weighing up to 40 kg. In certain species the cones are brightly coloured, possibly to attract animal pollinators and seed dispersers (Norstog and Nicholls, 1997). Pollen cones can take several months from emergence to progress to pollen shed, e.g. four months in *Encephalartos altensteinii* (Xaba, P., pers. comm).

According to Fernando et al. (2010) there is considerable diversity in male gametophytes amongst the four orders of gymnosperms (Cycadales, Ginkgoales, Coniferales and Gnetales). The differences occur in pollen and pollen tube morphology, development and cell composition (Yatomi, et al., 2002; Fernando, et al., 2005; Williams, 2008), sperm delivery (zooidogamy and siphonogamy) (Norstog, 1977) and the duration of pollen germination and fertilization (Chamberlain, 1935; Donaldson & Bösenberg, 1995; Norstog & Nicholls, 1997). Despite these differences, cycad pollen still possesses traits similar to anemophilous germplasm in that: a) it lacks a pollenkitt, a liquid fatty substance on the covering pollen grain that attracts insects in entomophilous angiosperms; b) most have an orbicule, which is a small acellular structure of sporopollenin (Pacini et al., 1999); and c) it has a multi-layered, thick sporoderm (Pacini, et al., 1999), which enables it to withstand desiccation, possibly as a result of this gymnosperm’s prolonged pollination period.

**Fig. 1:**
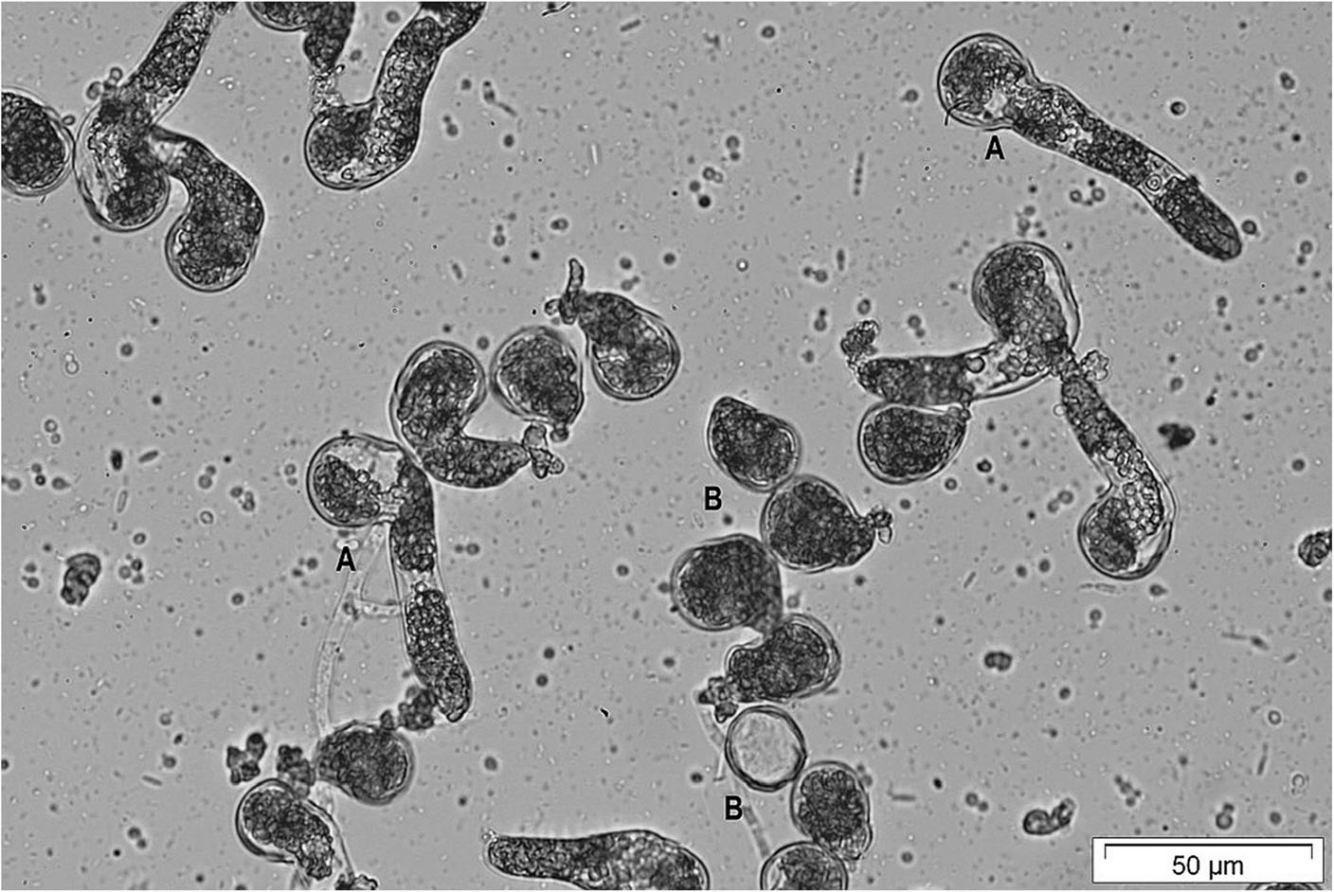
*Encephalartos latifrons* pollen germinating in vitro after 48 h incubation. A = pollen grains at various stages of tube growth and elongation; B = non germinated pollen (Xaba, 2014)

The similarities of entomophilous cycad pollen traits with that of anemophilous gymnosperms suggest that cycad pollen is robust, and well adapted to cope with detrimental environmental conditions. Various studies on pollen morphology in a number of South Africa cycad species revealed similarity in respect of shape and surface sculpturing between species unlike in any other group of plants (Marshall et al., 1989). Cycad pollen ranges in size, being c. 11 μm in *Macrozamia miquelii*, 23 μm in *Ceratozamia kuesteriana* (Dehgan and Dehgan, 1988), and 24.9 μm in *Cycas circinalis* (Raju and Rao, 2011). Fully hydrated pollen of *Encephalartos latifrons* is globular in shape and has grains of about 100 μm in diameter (Fig. 1).

Cycad pollen goes through nine developmental stages leading eventually to two spermatozoids (Singh, 1978; Ouyang et al., 2004). These spermatozoids are released from the pollen tube sac into the pollination chamber, following which one swims to fertilise the egg cell nucleus and the other fuses with the egg cell. The process is termed as single fertilization (Gifford and Foster, 1988; Chamberlain, 1935; Stanley and Linskens, 1974). This contrasts with angiosperms, which undergo double fertilization. *Microcycas* is the only known cycad that develops 16 spermatozoids and this is thought to be an ancestral characteristic (Norstog & Nicolls, 1997) not a derived trait (Nostog et al. 2004).

The entire process, from the deposition of pollen on the micropyle, followed by pollen tube growth and fertilization of the egg, takes considerably longer in cycads and other gymnosperms than in angiosperms. Pollination may occur three to seven months prior to fertilization. For example, in *Encephalartos altensteinii* it takes between 99 and 120 days from pollination to fertilization (Donaldson & Bösenberg, 1995). In contrast, fertilization intervals in angiosperms are usually a matter of hours (Norstog & Nicholls et al., 1997) or minutes (Williams, 2008).

Although all gymnosperm pollination was thought to be anemophilous (i.e., wind pollinated), pollination of cycads by insects (entomophily) has been reported widely (Stevenson et al., 1998) and proven experimentally in seven out of 10 extant genera: *Bowenia, Cycas, Encephalartos Lepidozamia, Macrozamia, Stangeria* and *Zamia* (Xaba, 2014).

Reproduction by individual plants can be annual, but may be sporadic or in intervals of up to or > 15 years, depending on species and environmental conditions (Liddle, 2009). For example in *Dioon edule* coning frequency is 3–9 years and 10–52 years in male and female plants respectively (Vovides, 1990). In nearly all cycads there is a marked and regular periodicity in cone initiation and development; this cycle can be retained even when the specimens are transported from the wild, often into different latitudes, hemispheres and climates. Many species tend to initiate cones in the spring, are pollinated in the summer and set seeds in late autumn or winter (Norstog and Nicholls, 1997). However, there are marked differences between genera and between species in the same genus with respect to the timing of their reproductive phases. For example, *Zamia integrifolia* develops cones in the autumn and is pollinated in December, but these stages occur several weeks later in north Florida than they do in the Miami area, 500 km to the south (Norstog and Nicholls, 1997). One factor for this may be that a more temperate-zone environment (e.g., thermo- or photo-period) may inhibit, or at least not stimulate, ‘cone’ induction. This area of cycad biology is not well studied and offers the opportunity for field and laboratory-based research.

During pollen shedding in cycads, the sporophylls on the pollen cone loosen to expose pollen sacs, which eventually dry out to liberate pollen grains into the atmosphere (Chamberlain, 1935). During this period, the sporophylls in some section of the seed ‘cones’ typically separate for a number of days to allow insect vectors and the transfer of pollen directly into the vicinity of the micropyle (Donaldson, 1997). A number of cycad species have been observed to have different lengths of cone opening, or receptive, periods. For example, *Encepalartos inopinus* cones are reported to be receptive for only four days (Grobbelaar, 1996). The second phase of pollen development begins after pollen is carried to the ovule and is drawn into the pollen chamber by resorption of a pollination droplet. This is typical of all gymnosperms (Chamberlain, 1935). Pollination drops have multiple functions: influencing pollen germination and tube growth; defending the ovule from pathogens; providing a food reward to insect pollinators. Proteins of the pollen drop in cycads represent the degradome and generally contain higher proportions of proteins localised to intercellular spaces (Prior, 2014).

One feature of pollen cone biology that has received considerable attention is thermogenesis. Since Poisson’s (1878) early study much evidence has emerged to support this (Jacot-Guillarmod, 1958) with the most extensive evidence showing thermogenesis in 42 out of 43 species tested across ten genera (Tang, 1987). Thermogenesis is mostly associated with insect, especially beetle, pollination where volatile odours as the result of cone heating could indicate food, mating opportunities and heat resources (Thien et al., 2000; Terry et al., 2004; Suinyuy et al., 2010; Tang, 1987). Since thermogenesis can influence the timing of insect movement between cones, it is reasonable to assume that receptive female cones, and especially the presence of pollination droplets, would be synchronised with periods of heating and insect dispersal from male cones.

### Storage

Because cycads are dioecious the collection, storage and creation of pollen banks for medium to long-term preservation and germplasm exchange is highly desirable. Indeed, artificial pollination may be necessary in cycad ex situ collections to ensure the setting of viable seed (Tang, 1986). The habitat and location of ex situ collections may mean a lack of suitable pollinators. In addition, such collections typically have limited numbers of each plant species. To counteract such a limitation, and based on microsatellite marker assessment of nearly 600 plants of *Zamia decumbens*, Griffith et al. (2015) recommended that collections can better conserve the genetic diversity of in situ populations as long as multiple accessions are made over more than one year and the following two approaches are taken: 1) species biology information informs the collecting strategy; 2) each population is managed separately.

Chamberlain (1926) reported that cycad pollen could last about a month in ambient atmospheric conditions but recent studies showed that different cycad genera varied in responses to similar storage environments (Tang, 1986). Furthermore, Mostert (2000) showed that pollen within the *Encephalartos* species differs in its ability to retain viability at sub-zero storage temperatures. Interestingly, Osborne et al. (1992) reported an anomalous cyclic viability in *C. thouarsii* pollen, where the pollen showed a dormancy-like phenomenon when tested every six months over a three years period. Osborne et al. (1991, 1992) also reported that *Encephalartos* pollen could be stored for three to five years at -15 °C.

Artificial pollination using short- to longer-term stored pollen may also be important in in situ conservation projects where plants are too far apart for pollination to occur, as in *E. latifrons* (Daly et al., 2006) and *E. middelburgensis* (P. Xaba, SANBI, RSA, pers. comm.) or where no pollinators are present. The two main methods for artificial pollination of cycads involve the injection of pollen between the loose sporophylls by puffing (dry method) or squirting in an aqueous solution (wet method) (Grobbelaar, 2002). The most efficient pollination methods may vary between species and dry pollination is recommended for *Bowenia, Ceratozamia, Chigua, Cycas, Dioon, Microcycas, Stangeria* and *Zamia*; wet pollination is suggested for *Encephalartos, Lepidozamia* and *Macrozamia* (Grobbelaar, 2002). However, *E. ferox* had more viable seed (82%) using a dry method (Tang, 1986). Xaba (2014) observed that seed germination and embryo presence was greater in *E. altensteinii* than *E. latifrons* irrespective of the use of dry or wet pollination. Interestingly, wet pollination in *E. latifrons* resulted in significantly lower seed germination and fewer embryos compared with the dry method, indicating potentially critical relations between pollen viability and environmental conditions. In orchids, humidity levels can impact on both lifespan and immediate viability, e.g., following the imposition of desiccation stress (Marks et al., 2014). Evidence of drying stress has also been seen in *E. latifrons* pollen after 24 h and 15 days of treatment with silica gel; in contrast, *E. altensteinii* pollen retains >60% germination after these treatments (Xaba, 2014). Similar pollen research is needed for a range of cycad species.

Whilst cycad pollen banks have been established by a number of botanic gardens, research on pollen germination and storage remains limited for most cycad groups. Osborne et al. (1992) established that stored pollen can be used to give consistent fertilization outcomes, demonstrating that asynchronous coning in cycads can be surmounted as pollen retained 50% viability for up to two years when stored at 0 °C. Similarly Xaba (2014) noted considerable success in storing silica gel dried pollen of 17 species of *Encephalartos* at -15 °C over a three year period. Over this storage period, there was no obvious difference between the remaining viability of *E. latifrons* from both wild and cultivates sources (Xaba, 2014). For the same lots of pollen, viability loss over 8 years followed a sigmoidal pattern (Xaba, 2014) typical of other pollens (e.g., Marks et al., 2014). Huang (2011) stored pollen of *C. revoluta* at −80 °C for 500 days without obvious loss of viability. Studies on *Cycas* species by Yang et al. (2009) involved the testing of various media and storage conditions. Storage temperature affected pollen viability, which was retained for 4 months at 0 °C; pollen viability did not show a significant loss after preservation in liquid nitrogen at a water content of 13.2–15.5%. Cryostorage is an accepted approach for many groups of species, such as fruit trees and orchids (Ajeeshkumar and Decruse, 2013), and may be an option for cycads as cryopreserved pollen of *C. elongata* yielded 90% seed set (Yang et al., 2009). However, cycad conservation and sustainable use through pollen preservation is hindered by limited research and baseline data on reproductive biology, pollen storage behaviour and in vitro manipulations to assess viability.

### Germination

As for other species, cycad pollen germination and pollen tube growth is influenced by several environmental factors, particularly temperature and moisture (relative humidity), (Stanley and Linskens, 1974). In in vivo environment, such effects are realised through a combination of external ambient conditions and the internal temperature of the cone as affected by thermogenesis when receptive.

Pollen viability / germination can be assessed in vitro by mixing the pollen with a solution comprising 10% (*w*/*v*) sucrose and 0.005% (w/v) boric acid (Osborne et al., 1992; Mostert, 2000) at a ratio of 20 mg pollen to 200 μL medium. Then a 15 μL droplet is suspended upside down on a Petri dish lid over water to prevent drying out (Xaba, 2014). After 48 h at 28 °C in the dark, the pollen drop is diluted and tube emergence assessed under a microscope (Fig. 1). For fresh pollen of *E. altensteinii* and *E. latifrons* in vitro germination can be around 80% (Xaba, 2014).

Pollen germination (in all plant species) is highly temperature sensitive. In cycads, evidence suggests that the optima are 15–35 °C for *E. altensteinii*, but only between 25 and 30 °C for *E. latifrons* (Xaba, 2014). The stricter temperature requirement for pollen germination in the latter species provides a much smaller window of opportunity for efficient pollen tube germination and growth, due to the restricted time that the ovulate cone temperature in the natural environment overlaps with this temperature zone (Xaba, 2014). At least for the two species mentioned above an in vitro pollen quality assessment at 28 °C appears to be appropriate.

Pollen viability testing using stains has also been carried out with nitroblue tetrazolium stain (Tang, 1986); acetocarmine and Alexander’s stain (Mostert, 2000) and aniline blue (Kay et al., 2011). In addition, Osborne et al. (1992) showed best results for cycad pollen germination using the hanging-drop assay with 15% sucrose solution with 0.005% boric acid at 28 °C and incubated for 48 h. Later Moster (2000) found that 5 and 10% sucrose, with a similar concentration of boric acid, yielded comparable results.

## Seed Biology for Conservation and Use

Whilst much is known about the seed biology of gymnosperms in general, little is known about the seed biology of cycads, either for storage or germination. Moreover, autecology studies tend to focus on plant characteristics, such as coning time, rather than seed traits (mass, storage, germination). Nonetheless, seed yield has been recorded in some species. For example in *Dioon edule*, mature female cones weigh around 2.5–5 kg and yield about 80–230 seeds (Vovides, 1990). One estimate indicates that *Zamia amblyphyllidia* yields only 15 seeds per cone (Negron-Ortiz et al., 1996).

Seed morphological features of cycads have been investigated in a few species. Mature seeds of five *Cycas* species (*C. revoluta, C. media, C. normanbyana, C. taiwaniana* and *C. wadei*) have a three-layered coat consisting of the sarcotesta, sclerotesta and a thin membraneous jacket (Dehgan and Yuen, 1983). The nutritional value of starch in the sarcotesta provides an incentive for local land dispersal by mammals, for example *E. whitelockii* seed by baboons and monkeys (Kamoga, D., pers. comm). Seeds of *Cycas* species in the section *Rumphiae*, such as *C. circinalis* and *C. thouarsii*, have an additional spongy layer that causes flotation and could enable long-distance dispersal by river or ocean currents. Seeds of species in this taxonomic section tend to be large and buoyant regardless of viability, whilst seeds in other taxonomic sections in *Cycas*, such as section *Asiorientialis*, containing the well-known species *Cycas revoluta*, have relatively small seeds that only float when inviable.

In cycads, small is a relative concept, as seeds are often 15–50 mm long (Table 2) and seed weights are all relatively large, from 1 to 25 g: *Bowenia spectabilis* (1.2 to 2.3 g), *Macrozamia spiralis* (3.2 g), *Cycas thouarsii* (6.7 g), *Encephalartos hildebrandtii* (7 g), *Macrozamia dyeri* (16 g), *Lepidozamia hopei* (18 g), *Macrozamia fraseri* (19 g) and *Cycas rumphii* (25 g) (Seed Information Database; http://data.kew.org/sid/). In other angiosperms, particularly tropical trees, the larger the seed mass the greater the likelihood that the seed is desiccation sensitive (Dickie and Pritchard, 2002; Daws et al., 2006).

**Table 2:** Morphological features of seed lots of seven cycad species

Species name	Seed size (mm) (mean ± SD) (*n* = 30)*		Embryo length (mm) (mean ± SD) (n = 30)*	Seed with embryos (% ± SD) (n = 30)*	Embryo: Seed length ratio (n = 30)*	Reference
	Length	Width				
*Cycas circinalis*	25–38	20–24	–	–	–	Raju and Rao (2011)
*Cycas revoluta*	36.4 ± 5.2	27.1 ± 2.1	12.1 ± 0.5	53.2 ± 0.2	0.33	Dandugula (2011)
*Cycas siamensis*	34.0 ± 4.1	23.7 ± 1.1	10.8 ± 1.0	82.3 ± 2.45	0.32	Umair (2011)
*Cycas taitungensis*	49.79 ± 2.55 (n = 20)	32.32 ± 1.69 (*n* = 20)	8.50 ± 0.45 (*n* = 5)	–	0.17	Chien et al. (2012)
*Dioon edule* (Lot 1)	22.7 ± 6.4	18.6 ± 3.5	12.5 ± 1.1	67.1 ± 1.55	0.55	Dandugula (2011)
*Dioon edule* (Lot 2)	21.7 ± 1.4	19.1 ± 2.5	11.8 ± 3.4	6.6 ± 2.50	0.54	Dandugula (2011)
*Zamia furfuracea*	15.0 ± 4.6	10 ± 1.1	10.8 ± 1.0	82.3 ± 7.51	0.72	Umair (2011)
*Zamia floridana*	18.1 ± 6.2	11.5 ± 3.2	12.5 ± 1.1	67.1 ± 1.26	0.69	Umair (2011)

*n = 30, except where it is indicated in the bracket

### Storage

Globally, cycad conservation through seed storage presents complex challenges. Knowledge of seed biology and storage behaviours is limited for most cycad taxa, compared with other plant groups. The Seed Information Database (http://data.kew.org/sid/) only lists seed storage biology data for four *Cycas* species and one species each in *Encephalartos* and in *Macrozamia*. The compiled botanical information on cycad seed storage covers <2% of species in the Cycadales.

*Cycas angulata*, *C. armstrongii*, *C. media* and *Macrozamia reidlei* germinated after sun-drying (Langkamp and Plaisted, 1987) and the possibility exists that the seeds could be orthodox in their storage response, i.e., the seeds can tolerate rapid artificial drying to 15% RH and storage at −20 °C. This remains to be established, however. For *C. revoluta* the seeds had 92% germination after 6 months air-dry storage at 2 °C, compared with 42% germination when stored open at 22 °C (Dehgan and Schutzman, 1989). This suggests that seeds of this species are not recalcitrant (i.e., not highly desiccation sensitive). *E. natalensis* seeds also seem to be quite robust in storage, as Forsyth and van Staden (1983) report that seeds can be stored dry or moist. Similarly, *Zamia integrifolia* is reported to have 77% germination after 1 year air-dry storage at 5 °C (Witte, 1977).

Overall then, the main restriction to developing a strategy on the ex situ conservation of cycads in seed banks is a lack of baseline data on storage behaviour, combined with challenges in securing adequate seed supplies, especially in the case of rarer species.

### Germination and Dormancy

The level and timing of seed germination in cycads varies between species. It has been observed that seeds of *Microcycas* and *Cycas revoluta* sometimes begin germination even while in the cone, and less than three to four months after fertilisation (Chamberlain, 1935, Norstog and Nicholls, 1997). In contrast, *E. latifrons,* a critically endangered cycad propagated at Kirstenbosch National Botanical Garden (KNBG), shows low seed germination (Xaba, 2014). Generally, cycad seed is sown to germinate around 25–30 °C (Table 3). This is true for *C. revoluta* (Frett, 1987) and *Zamia* seed (Witte, 1977), although lower temperatures of 15–20 °C have also been reported for *C. revoluta* (Zarchini et al., 2011).

**Table 3:** Germination conditions and responses for eight species of cycads

Species name	Germination (Mean ± SD) (*n* = 100)	Seed pre-treatment	Germination		Reference
			T (°C)	Substrate	
*Cycas revoluta*	100 ± 0	Seed coat removed	25	Water-agar	Dandugula (2011)
*Cycas siamensis*	100 ± 0	–	25	Water-agar	Umair (2011)
*Cycas taitungensis*	75–84	–	30/20 and 20	Moist sphagnum moss	Chien et al. (2012)
*Dioon edule*	100 ± 0	–	25	Water-agar	Dandugula (2011)
*Encephalartos altensteinii*	c. 60% in 30 weeks	Ambient storage, 8 months;	28	Silica sand	Xaba (2014)
*Encephalartos latifrons*	c. 50% in 30 weeks	Ambient storage, 8 months;	28	Silica sand	Xaba (2014)
*Zamia floridana*	100 ± 0	–	25	Water-agar	Umair (2011)
*Zamia furfuracea*	100 ± 0	–	25	Water-agar	Umair (2011)

Slow germination is known to be a feature of many cycads. Frett (1987) observed that *Cycas revoluta* seeds take up to a year to germinate: removal of the pulp and sowing in the dark have been observed to increased germination after scarification (up to 2 h) with sulphuric acid, but the application of gibberellic acid, a plant hormone and well known dormancy-breaking chemical, does not appear to provide any enhancement. There is some evidence that species with a thick sclerotesta (*C. revoluta, C. circinalis, Zamia floridana*, *Z. furfuracea*) may be rather impermeable to water (Dehgan, 1996). Consequently, sulphuric acid digestion has been tried – a 25% concentration for 2 h increased germination rate in *C. revoluta* (about 30% earlier; 230 days vs 310 days); however, overall germination was lower than fresh seed (Zarchini et al., 2011). This indicates that care must be exercised when using acid treatments that might weaken the seed coat, therefore, chemical contact with the embryo should be avoided to minimise viability loss. Other forms of scarification may improve germination, such as the mechanical treatment (sanding down) of the coronula at the apex of the seed kernel, which is effective on the hard seeds of *Dioon merolae* (Perez-Farrera et al., 1999) and *Encephalartos poggei.* (Jones, 2002). Scarification by wet heat (i.e., hot water), can reduce the total germination in *Cycas revoluta* seeds (Zarchini et al., 2011).

In angiosperm seeds, slower germination is often observed when the embryo is relatively small, and might indicate the presence of some type of dormancy. Dormancy is the inability of the seed to germinate in a specified period under any combination of normal physical environmental factors that are otherwise favourable for its germination, i.e., after the seed becomes non-dormant (see Baskin and Baskin, 2014; Finch-Savage and Leubner-Metzger, 2006). Cycads are well known to have a very slow embryo development (Gifford and Foster, 1988; Singh, 1978) and seeds of most cycad species are dispersed whilst the embryos are relatively small. Subsequent slow germination in some cycads may reflect the need to complete embryo development and could indicate the presence of morphological or morpho-physiological dormancy. For example, embryo length relative to the seed (E:S ratio) varies considerably, from 0.17 in *Cycas taitungensis* to about 0.7 in *Zamia furfuracea* and *Zamia floridana* (Table 2). It appears that once the embryo has reached more than 50% of the length of the seed germination can be relatively rapid, e.g., within c. 30 days (Lindstrom, A., pers. comm). Relative embryo size can be a potential indicator of habitat and environmental stress tolerance in Apiaceae; a positive correlation was found between relative embryo length with germination speed, and negative correlation with the amount of habitat shade (Vandelook et al., 2012). Moreover in the family Grossulariaceae, species in the genus *Ribes* that disperse seeds with larger relative embryo lengths germinate more easily (Mattana et al., 2014). Similarly, there seems to be a correlation between initial relative embryo length at ripening and the natural habitat where the species occurs, e.g., distinct tropical species, such as *E. ituriensis*, germinate immediately at ripening while most South African *Encephalartos* need at many months storage prior to sowing and germination. For example, seeds of *C. revoluta* (Frett, 1987) and *E. latifrons* and *E. altensteinii* (Xaba, 2014) benefit from storage at room temperature for 6–12 months, after which they germinate faster than fresh seeds.

One recognised problem during cycad seed germination testing is that sowing seeds with small embryos immediately after harvest results in water uptake and expansion of the gametophyte and the opening of the sclerotesta. The exposed gametophyte tissue then often succumbs to fungal infections before the embryo has grown large enough to germinate.

Whilst the optimum temperatures for germination have been characterised for a wide range of seed species (Durr et al. 2015), detailed data on cycad seed temperature sensitivity is not yet available. Moreover, many questions remain unanswered concerning the nature of any dormancy present. These topics should be the focus of future research.

## Tissue Biology

Cycad conservation could potentially benefit from the application of in vitro technology and micropropagation (tissue culture), cryopreservation of cells and tissues and other innovative technologies, including molecular biology. Micropropagation may also support sustainable horticultural practices and the production of disease-free genetic resources. A knowledge of seed biology, phenology and seed and pollen storage behaviour will be pivotal to the application of conservation biotechnology to cycads. Cases in point are the use of seed-derived explants to initiate tissue cultures and the respective storage of zygotic and somatic embryos in traditional seed banks and in vitro genebanks in which tissue cultures are maintained in medium and long-term (cryogenic) storage (Litz et al., 2004). In this regard there has been some progress in recent years on the in vitro growth of cycad tissues (Teixeira da Silva et al., 2014).

### Marker Technologies

Harding and Benson (2012) have recently reviewed the use of biomarkers in tropical plant conservation. With respect to cycads, molecular-biotechnological approaches can be applied in seed biology research and to facilitate the development of storage practices. Population genetics and taxonomic studies have applied cycad biomolecular markers (chloroplast and nuclear DNA sequence variation; ITS, ISSR, RAPD, SSR markers and AMOVA analysis) to study population structures and phylogenetic relationships. Calonje et al. (2011) used molecular techniques to develop and inform genetics-based conservation management and action plans for *Zamia lucayana,* the only cycad species endemic to the Bahamian archipelago, the habitats of which are at risk from urban development. In their study, sixteen microsatellite DNA markers were used to determine the number of management units required and to assess genetic structures of known populations. More recently single-copy nuclear gene technology has been used to describe the phylogeny of cycads, contributing to an accurate infra-familial classification of Zamiaceae (Salas-Leiva et al., 2013). Rousseau (2012) took a molecular systematic approach to 63 *Encephalartos* species to barcode various DNA regions (matK, rbcLa, psbA-trnH, and nrITS), finding that genetic variation was extremely low as was resolution at species level. Undoubtedly, DNA barcoding programmes do have the potential to support related cycad conservation activities (Chaw et al., 2005; Sass et al., 2007; Little and Stevenson, 2007). But to support law enforcement in the illegal cycad trade, modern technologies will need to provide unequivocal identity confirmation. The use of micro-chipping technology to tag cycads has had success in apprehending poachers / collectors acting illegally. Also, forensic chemistry of cells and tissues, pioneered on tropical timber, has been used to compare isotope signatures of wild cycads with specimens translocated decades ago (Nordling, 2014). This approach was successfully applied to two species in *Encephalartos* (*E. lebomboensis* and *E. arenarius*) by determining various isotopes (carbon, strontium, nitrogen, oxygen, sulphur) in the tissues (Retief et al., 2014). The prospect of identifying cycads growing ex situ that have been illegally traded is being explored further.

With respect to reproductive biology, 16S rRNA mitochondrial genes have been used to study the lineages of weevils involved in cycad pollination (Tang et al., 2011). Biochemical markers (total protein, isozymes) and molecular markers (RAPDs, ISSR) have been tested to establish early sex differentiation to facilitate cycad conservation and sustainable management. Transcriptomics is also used for characterisation of male and female plants in *C. elongata* (Wang et al., 2011). Genes related to phytohormones and volatiles seems to suggests a complex molecular basis for sexual differentiation in cycads and this information could be used as biomarkers for sexual identification. Genes related to DNA methylation were also found to be associated with sexual differentiation in *Cycas elongata,* indicating the potential use of epigenetic biomarkers for sexual identification (Wang et al., 2011)*.* Collectively these different molecular approaches may be used to support seed biology research and germplasm storage practices.

### Tissue Culture

The IUCN-SSC Cycad Action Plan (Donaldson, 2003) highlights the potential for using in vitro propagation and tissue culture to improve germination, increase growth rates of seedlings, and develop plants from tissue culture for species with low seed set, all of which contribute to having more plants in cultivation. However, there have been comparatively few successes propagating cycads through tissue culture compared to other plant taxa (Dhiman and Rautela, 2014), with reports on only 17 species, representing <5% coverage of species (Table 4). Genera of interest have been *Encephalartos* (five species), *Zamia* (four species), *Ceratozamia* and *Cycas* (three species each) and *Stangeria* (one species). A range of source tissues have used, particularly for studies involving *C. revoluta* (Teixeira da Silva et al., 2014; and references therein).

**Table 4:** Various explants used for cycad tissue culture and their regeneration

Explant type	Species	Types of regeneration (variable with species)
Megagametophyte	*Ceratozamia hildae, Ceratozamia mexicana, Cycas circinalis, Cycas revoluta, Dioon edule, Encephalartos altensteinii, Encepalartos umbeluziensis, Zamia fischeri, Zamia furfuracea, Zamia pumila* (syn. *Z. integrifolia*)	Callus formed; or coralloid roots developed after callus formation or from spherical outgrowths; or embryoids formed; or seedlings grew.
Zygotic embryos (usually cut transversally or longitudinally or into blocks)	*Cycas revoluta, Encephalartos cycadifolius, Encephalartos dyerianus, Encephalartos natalensis, Dioon edule, Zamia pumila* (syn *Z, latifoliata*)*, Zamia latifolia,*	Callus formation triggered with phytohormones; potentially leading to root or shoot or plantlet formation, preferentially from compact callus and somatic embryos (may require ABA for maturation).
Cotyledonary leaf sections or epicotyl sections from zygotic embryos	*Cycas revoluta*	Adventitious root and shoot formation medium- and phytohormone-dependent.
Cut, young leaf flushes of mature trees	*Ceratozamia euryphyllidia*	Somatic embryo production and plantlet conversion.
Seed cotyledon, plant scales and in vitro seedling shoot tips	*Cycas revoluta*	Shoot bud formation; or shoot tip growth.
Seedling epicotyl and hypocotyl (1 cm long)	*Cycas revoluta*	Callus proliferation only.
Root of seedlings	*Stangeria eriopus*	Production of compact white callus leading to leaf emergence.
Root or stem	*Cycas guizhouensis*	Callus formation.
Bulbil inner bulb-scales	*Cycas revoluta*	Shoot buds differentiated.

^a^Modified from Teixeira da Silva et al. (2014) where a full list of references is provided

Early studies resulted in the asexual production of embryos from explanted somatic and reproductive (zygotic embryos/seed) tissues of *Ceratozamia* and *Zamia* (Chavez et al. 1992 a, b, c). Moreover, a range of generally routine methods for the production of somatic embryos have been developed by Litz and colleagues (Chavez et al. 1992 a, b, c; Chavez et al., 1994; Litz et al., 1995 a, b) but these have not, generally, resulted in the growth of whole plants. Megagametophytes and zygotic embryos have been used in most studies and in some cases, organogenesis has been reported from cut zygotic embryos (Table 4).

The lack of proven methods for the long-term storage of cycad seeds has resulted in the suggestion that cryopreservation may be the way forward for the long-term storage of cycad material, i.e., in cryobanks (Litz et al., 2004; Wade et al., 2016). Certainly, improvements in cycad tissue culture will expand the options for cryopreserving somatic embryos and rescuing zygotic embryos post-cryo. Nonetheless, a cycad embryo cryopreservation method is yet to emerge. The development of in vitro methods for pollen germination will be a valuable adjunct to pollen storage studies, particularly as the successful cryopreservation of *C. elongata* pollen has been reported, achieving 90% seed set (Yang et al., 2009). This will help to elevate the problem of a-synchronised coning in male and female plants as the pollen can be stored and tested for viability before conducting an artificial pollination.

## Conclusions

Best practices and efficiency measures have been developed for cycad horticulture in botanic gardens (Cuestas et al., 2011), resulting in the development of high quality collections – particularly in South Africa, Thailand and China – that help to safeguard against species loss in the natural environment. Such losses might accelerate due to many risk factors, including climate change related movement of diseases and pests (Ma et al., 2011). The 2010 provisions of the IUCN-CSG Action Plan (Donaldson, 2011) consider cycad risk mitigation with an emphasis on phytopathological issues. In this respect in vitro genebanks and cryobanks offer the advantages of enabling phytosanitary control and conserving disease-free cycad germplasm. The ready availability of germplasm for ex situ conservation and sustainable use will only be realised, however, by exploring the comparative seed and pollen biology of a much wider range of species than hitherto studied. Not only will such investigations improve understanding of species’ physiological adaptations to the natural environment but also increase confidence in implementing assisted reproduction programmes for the most threatened cycads.
